# Exercise During Pregnancy: What Do OB/GYNs Believe and Practice? A Descriptive Analysis

**DOI:** 10.1089/whr.2021.0132

**Published:** 2022-02-28

**Authors:** Rachael K. Nelson, Selina M. Hafner, Alyssa C. Cook, Natalie J. Sterner, Erin L. Butler, Brooke E. Jakiemiec, William A. Saltarelli

**Affiliations:** Division of Exercise and Health Sciences, School of Health Sciences, Central Michigan University, Herbert H. and Grace A. Dow College of Health Professions, Mount Pleasant, Michigan, USA.

**Keywords:** exercise, reproductive health, cardiovascular health, gynecology

## Abstract

***Introduction:*** Although regular exercise is recommended during non-complicated pregnancies to promote maternal and fetal/infant health, estimates suggest that only 15% of expectant mothers achieve current exercise recommendations. Although lack of motivation and fear related to potential fetal injury are often cited as reasons for not engaging in regular physical activity/exercise during pregnancy, less is understood about individual attitudes and practice habits of obstetrician and gynecologists (OB/GYNs) regarding exercise recommendations that may influence patient engagement in exercise during pregnancy.

***Purpose:*** To describe the attitudes, knowledge, and clinical practice of OB/GYNs regarding exercise during pregnancy.

***Methods:*** Surveys were sent *via* U.S. mail to 950 practicing OB/GYNs identified *via* publicly available databases. The survey included 11 questions regarding demographic information, exercise physiology knowledge, as well as their attitudes and clinical practice recommendations regarding exercise during pregnancy.

***Results:*** One hundred thirty-nine completed surveys were returned (14.6% response rate). Ninety-four percent of physicians surveyed agreed that there are benefits of exercise during pregnancy and/or the benefits of exercise during pregnancy outweigh the risks. Ninety-eight percent of physicians surveyed reported that they (or their medical staff) routinely advise their patients to exercise during pregnancy and 46% reported discussing exercise guidelines related to time, intensity, and type of exercise. Only 13% of physicians surveyed reported taking a semester-long exercise physiology course, yet 27% of physicians surveyed reported developing personalized exercise prescriptions for all (6%) or some (21%) of their patients.

***Conclusions:*** Low exercise engagement among expectant mothers does not appear to be due to a lack of guidance or negative views of OB/GYNs regarding exercise during pregnancy.

## Introduction

Guidelines regarding exercise during pregnancy were established by the American College of Obstetricians and Gynecologists (ACOG) in 1985.^1^ However, these guidelines were largely restrictive and cautious due to a paucity of data demonstrating the safety and efficacy of exercise during pregnancy.^1^ As available data highlighting the benefits of exercise during pregnancy increased, reservations regarding exercise during preg-nancy were reduced, resulting in a major shift in 2002 when exercise guidelines for pregnancy matched that of the general population promoting accumulating at least 30 minutes of moderate-to-vigorous aerobic exercise most days of the week.^2^

The most recent opinion statement of the ACOG recognized that exercise/physical activity during pregnancy is associated with minimal risks (although some modification may be necessary due to normal gestational anatomical and physiological changes). Importantly, advancements in guidelines stem from a growing body of literature illustrating favorable maternal and fetal/infant health outcomes during pregnancy, at delivery, and postpartum associated with exercise during pregnancy.^3^ More specifically, regular physical activity/exercise during pregnancy is associated with lower gestational weight gain and risk of developing gestational diabetes among women who enter pregnancy overweight or obese.^4^

Moreover, exercise during pregnancy is associated with lower long-term risk of developing type 2 diabetes for mother and child.^5^ There are also cardiovascular benefits to regular exercise during pregnancy, with a lower incidence of gestational hypertension.^6^ Further, exercise during pregnancy is also associated with less lower back pain,^7^ increased energy levels,^8^ and improved mood^9^ that are indicative of a greater quality of life during the gestational period for these women.

Regarding labor and delivery, regular physical activity/exercise during pregnancy is associated with a lower risk of pre-term delivery,^10^ lower risk of macrosomia,^11^ and cesarean delivery.^6^ In regard to the postpartum period, women who engage in regular exercise during pregnancy also show a lower retention of pregnancy weight postpartum^12^ and lower incidence of postpartum depression.^13^ Importantly, many of the health benefits of exercise during pregnancy (*e.g.*, lower gestational weight gain, rates of hypertension, and gestational diabetes) are in the absence of additional lifestyle changes (*e.g.*, dietary modifications).^4,6,14^

However, despite the well-documented health benefits of exercise during pregnancy, estimates suggest that only 13%–43% of pregnant woman accumulate 20–30 minutes of aerobic activity most days of the week recommended by the U.S. Department of Health and Human Services and the American College of Obstetrics and Gynecology during uncomplicated pregnancies.^15,16^

Identifying the root cause of physical inactivity during pregnancy is complex due to a number of factors that influence exercise engagement in the general population that are further compounded by the added burden of pregnancy. For example, lack of time is often highlighted as one of the most common barriers to engagement in regular physical activity/exercise in the general population^17^ and among pregnant women.^18,19^ Unfortunately, time as a barrier to regular exercise during pregnancy is even more burdensome among expectant mothers who already have children due to childcare needs.^20^

Fatigue and lack of energy, only getting worse as pregnancy progresses, have also been identified as barriers to exercise during pregnancy.^21,22^ Importantly, women also report not knowing whether exercise is safe during pregnancy, what exercise recommendations during pregnancy are, and receiving conflicting information as additional barriers to exercise during pregnancy.^20,22,23^ To that point, there is little understanding regarding how physicians caring for women during pregnancy (*i.e.*, obstetrician and gynecologists “OB/GYNs”) view and prescribe exercise to their patients during pregnancy.

Understanding current perceptions and practices of OB/GYN's regarding exercise engagement during pregnancy is a vital step in developing and implementing effective intervention strategies to promote regular exercise during pregnancy. Therefore, the purpose of this study is to describe attitudes, knowledge, and practice behavior of OB/GYNs regarding exercise during pregnancy.

## Materials and Methods

### Study participants

A survey-based study was used to determine attitudes, knowledge, and clinical practice of 108 OB/GYNs regarding exercise during pregnancy from 13 states throughout the United States (Table 1). Participants had to self-report being ≥18 years of age and currently practicing OB/GYN in the United States to participate in this study. This study was approved by the Central Michigan Institutional Review Board.

**Table 1.** Survey

Content area	Questions
Demographics	What is your age? Biological sex? How many years have you been in practice? What U.S. state are you currently practicing in? What was your undergraduate major for your Bachelor's degree?
Knowledge	Have you ever taken a semester-long exercise physiology course? According to the ACOG, regular exercise is recommended for all women during uncomplicated pregnancies (True or False)
Attitude	Attitude regarding exercise during pregnancy (check all that apply)? I believe that exercise during pregnancy has risk. I believe that exercise during pregnancy has benefits. I believe that the benefits of exercise during pregnancy outweigh the risks. I don't believe that it is the physician's responsibility to prescribe exercise
Clinical practice	Do you routinely advise pregnant patients to engage in regular exercise? What categories of physical exercise do you or your staff discuss with patients? Do you or your staff work to develop a personalized exercise prescription for patients?

ACOG, American College of Obstetricians and Gynecologists.

### Survey method

The survey contained 11 questions: 4 demographic questions based, 1 question addressing exercise physiology coursework, and 6 questions related to the physician's overall views and clinical practices associated with advising patients on exercise during pregnancy (Table 1). One survey question specifically focused on identifying the areas of the “FITT Principle” that physicians discuss with their pregnant patients. Importantly, the FITT Principle provides a framework for describing exercise prescription parameters related to frequency, intensity, time, and type.^24^

Potential participants were identified online through practice websites and physician databases. Potential participants were sent a cover letter inviting them to participate in the study, consent document, survey, and a pre-paid, self-addressed envelope to return the survey. A total of 950 surveys (along with a cover letter and a self-addressed, stamped, return envelope) were sent *via* the U.S. postal service. To maintain privacy and confidentiality, potential participants were only contacted once to solicit participation. In addition, no personal identifiers were collected on the survey.

### Data analysis

Descriptive statistics including mean, standard deviation, and range were calculated for continuous variables (*i.e.*, age and years in practice). Frequencies were calculated for categorical variables (*i.e.*, sex, state of practice, undergraduate major, and all remaining questions related to attitudes, knowledge, and clinical practices regarding exercise during pregnancy). A Chi-squared test was used to examine potential differences in responses regarding exercise during pregnancy for OB/GYNs who had been in practice for ≥20 vs. <20 years. All statistical analysis was performed by using SigmaPlot 12.5, and statistical significance was set at *p* < 0.05.

## Results

One hundred thirty-nine completed surveys were returned for a response rate of 14.6%. Participant demographics are presented in Table 2. All participants (100%) agreed with the statement that “regular exercise is recommended for all women during uncomplicated pregnancies.” Regarding attitudes toward exercise during pregnancy, 94% of participants agreed that there are benefits to exercise during pregnancy and/or the benefits of exercise during pregnancy outweigh the risks. Regarding exercise recommendations during pregnancy, 97% of participants reported that they, or their medical staff routinely advise their patients to exercise during pregnancy.

**Table 2.** Participant Demographics

Variable	Count	Mean ± SD	Range
Sex (male/female)	54/85		
Age (years)		52.2 ± 9.9	32–71
Practicing (years)		21.7 ± 10.0	2–43
U.S. state currently practicing (*n*)			
Alabama	8		
Hawaii	7		
Illinois	6		
Indiana	9		
Iowa	10		
Kansas	3		
Michigan	13		
Minnesota	8		
Mississippi	5		
Missouri	8		
Nebraska	8		
North Dakota	16		
Ohio	11		
Pennsylvania	8		
South Dakota	10		
Wisconsin	9		
Undergraduate major (%)			
Biology	50		
Chemistry	9		
Other	41		

SD, standard deviation.

Further, 46% of physicians surveyed reported discussing exercise guidelines related to exercise intensity, time, and type. Although only 13% of participants reported taking a semester-long Exercise Physiology course, 30% of physicians surveyed reported developing a personalized exercise prescription with some (21%) or all of their patients (6%). The proportion of responses did not differ when stratified by years in practice (*i.e.*, ≥20 vs. <20 years of practice as an OB/GYN).

## Discussion

The purpose of this study was to gain a better understanding of the knowledge, attitudes, and clinical practice of OB/GYNs regarding exercise during pregnancy throughout the United States. To that point, we found that an overwhelming majority of OB/GYNs who participated in this study consider exercise to be beneficial during pregnancy and they often advise their patients about exercise during pregnancy. However, we found low rates of physicians discussing specifics regarding guidelines for exercise (*e.g.*, time, intensity, and type) or personalized exercise prescriptions with their patients during exercise.

Encouragingly, all the OB/GYNs who participated in this study agreed that regular exercise should be recommended for women undergoing uncomplicated pregnancies. Guidelines regarding exercise recommendations during pregnancy have shifted since they were initially released by the ACOG in 1985.^1^ Initial guidelines for exercise during pregnancy were originally restrictive and cautious due to a paucity in available data regarding safety and efficacy.

By 2002, guidelines for exercise during pregnancy matched guidelines for the general population.^2^ Consequently, later reports indicated that 89% of health care providers believed that sedentary women could safely start exercising during pregnancy.^25^ Further, 98% reported that current exercisers should continue to exercise throughout pregnancy.^25^ Importantly, this is in support of existing data highlighting exercise as a protective factor for human health and well-being.^4–7,9–13^ To that point, the ACOG recommends that pregnant women exercise at least 150 minutes a week.^3^

Even women who were not previously active before pregnancy are encouraged to participate in light exercises such as walking and yoga.^26^ Importantly, even low-intensity exercise has been shown to reduce pregnancy pain and improve the overall experience of pregnancy for women both physically and mentally.^26,27^ Despite all the aforementioned support of women exercising during pregnancy, most women do not meet current guidelines for exercise during pregnancy.^28^

We found that the OB/GYNs surveyed in this analysis, regardless of years in practice, reported that exercise during pregnancy carries benefits and/or the benefits of exercise during pregnancy outweigh the risks. Although some participants agreed that there are risks and benefits to exercise during pregnancy, the majority agreed that the benefits of exercise during pregnancy outweigh the risks. Importantly, no participants reported only risks associated with exercise during pregnancy.

This is consistent with previous reports that also found that 98%–99% of physicians surveyed agreed that exercise during pregnancy is beneficial.^25,29^ However, women still report concerns regarding the risk associated with exercise as one of the primary reasons why they refrain from exercise during pregnancy.^20^ This may be due to changes in guidelines and physician practices. Before the 2002 ACOG guidelines for exercise during pregnancy, typically only women who were regular exercisers before pregnancy were encouraged to continue engaging in exercise during pregnancy. However, the 2002 ACOG guidelines encouraged nearly all women to exercise during pregnancy, not just those that were regular exercisers before pregnancy.^2^

However, when stratified by years in practice, we did not observe differences regarding the associated benefits/risks during pregnancy and years in practice (*i.e.*, ≥20 vs. <20 years of practice as an OB/GYN). Our data underscore that physicians know and understand the benefits of exercise during pregnancy. What we do not know is whether physicians are discussing the overall positive benefits of exercise with their patients and what patients perceive hearing. Importantly, Fredriksson et al. observed a strong correlation between an individual's level of knowledge on the benefits of exercise and that person's activity level.^30^

Collectively, this suggests that a lack of information given to patients about the benefits of exercise during pregnancy could explain, at least in part, why engagement in recommended exercise is so low during pregnancy. In addition, an increase in information regarding the benefits of exercise for expectant mothers may be an effective intervention strategy for increasing exercise engagement during pregnancy. A recent investigation found that providing educational information regarding exercise during pregnancy (in the form of a brochure), in conjunction with access to a fitness facility, was effective at reducing the time engaged in sedentary behavior among pregnant women in a rural community.^31^

However, the impact of providing educational information on the benefits of exercise during pregnancy alone on exercise behavior during pregnancy remains unclear.

Although expectant mothers still report a lack of knowledge regarding exercise guidelines during pregnancy as a barrier to exercise,^20^ the results of our survey indicate that nearly half of the OB/GYNs surveyed reported discussing all three components of the FITT Principle (*i.e.*, frequency/time, intensity, and type) with their patients. Interestingly, exercise modality or type (*e.g.*, walking vs. elliptical) was the most frequently reported principle discussed with patients.

This may be due to potentially discouraging certain exercise modalities that during pregnancy related to safety concerns such as losing balance (*e.g.*, road biking). The least reported principle was time. One-third of OB/GYNs surveyed in our study reported going a step further and proving patients with personalized exercise prescriptions. The details of that personalized exercise prescription remain unknown. However, the specifics of the individualized exercise programs may not be as important as the individualized exercise prescription itself. For example, available evidence indicates that individualized exercise prescription plans result in greater health outcomes and exercise adherence than standardized exercise prescription programs.^32^

With only one-third of OB/GYNs survey in our study reporting discussing personalized exercise prescription plans with their patients, there is room for improvement and perhaps referral to an Exercise Physiologist. Our findings are similar to previous reports suggesting a lack of exercise guidance from physicians to patients regarding exercise during pregnancy.^29^ A personalized exercise prescription plan takes additional time that an OB/GYN may be unable to provide their patients.

In addition, given that only a small number of patients (13%–43%) meet current exercise guidelines, it may discourage physicians from spending additional time developing individualized exercise prescriptions plans if most patients are unable to follow through. Shortness of office visits may also be a contributing factor. Many OB/GYNs feel other health-related matters are more essential and prioritize them over physical activity recommendations.^33^

Confounding matters is that reports indicate that advice from OB/GYNs regarding exercise is not only limited, but it can also be inconsistent.^33^ Therefore, referring patients to an exercise physiologist knowledgeable regarding exercise guidelines and safety measures during pregnancy could save physicians time and increase exercise participation during pregnancy.

There are certain limitations to the current study. Importantly, there is a potential for response bias in the current analysis. More specifically, it is possible that OB/GYNs who regularly discuss exercise with their pregnant patients, are interested in the topic, or even exercise themselves may be more likely to respond to this survey. This would suggest higher response rates for outcomes related to exercise prescription behavior in clinical practice.

Future studies are needed to minimize/eliminate response bias to obtain a more universal understanding clinical practice regarding exercise during pregnancy. Another limitation of the current analysis is the brevity of the survey, which results in a lack of detailed responses. However, the goal of our analysis was to describe the knowledge, attitudes, and clinical practice of OB/GYNs regarding exercise during pregnancy and to that point were able to gain a better understanding of all three and lay the foundation for more detailed follow-up studies.

There were also certain assumptions in the design of our survey. More specifically, we asked respondents whether they had ever taken a semester-long exercise physiology course as a potential indicator of exercise prescription knowledge/familiarity. However, an element of heterogeneity exists in college curriculum, and it is not a guarantee that exercise prescription and/or exercise prescription for special populations (*e.g.*, pregnancy) would have been covered in that coursework.

Finally, the results of this survey do not represent all U.S. states.^19,25^ However, this was more representation that previous reports focused on just one state. In addition, the results of our analysis represent diverse regions throughout the United States, including states with high and low ratings for health care for women.

## Conclusions

In summary, the results of our survey illustrate a widely held understanding among OB/GYNs that exercise during pregnancy is beneficial (or that the benefits of exercise during an uncomplicated pregnancy outweigh the risks). Further, we found that the vast majority of OB/GYNs are promoting the engagement of exercise during pregnancy with their patients and some even take the time to develop individualized exercise prescription plans for their patients.

Despite their efforts, the reports of women meeting current exercise recommendations during pregnancy still remain low. The potential areas for increasing exercise engagement activity during pregnancy include: disseminating information regarding the health benefits of exercise during pregnancy for expectant mothers and children, development of individualized exercise prescription plans for patients, and a referral system to exercise physiologist knowledgeable regarding exercise guidelines and safety measures during pregnancy.

Providing these resources to pregnant women has the potential to draw more women to a physically activity lifestyle during pregnancy, potentially impacting long-term health for patient and child.

## Abbreviations Used

ACOG: American College of Obstetricians and Gynecologists
OB/GYNs: obstetrician and gynecologists
SD: standard deviation
